# A fast exact sequential algorithm for the partial digest problem

**DOI:** 10.1186/s12859-016-1365-2

**Published:** 2016-12-22

**Authors:** Mostafa M. Abbas, Hazem M. Bahig

**Affiliations:** 1 Qatar Computing Research Institute, Hamad Bin Khalifa University, Doha, Qatar; 2 Computer Science Division, Department of Mathematics, Faculty of Science, Ain Shams University, Cairo, 11566 Egypt

**Keywords:** Restriction site analysis, Digestion process, Partial digest problem, DNA, Bioinformatics algorithm, Breadth first search

## Abstract

**Background:**

Restriction site analysis involves determining the locations of restriction sites after the process of digestion by reconstructing their positions based on the lengths of the cut DNA. Using different reaction times with a single enzyme to cut DNA is a technique known as a partial digestion. Determining the exact locations of restriction sites following a partial digestion is challenging due to the computational time required even with the best known practical algorithm.

**Results:**

In this paper, we introduce an efficient algorithm to find the exact solution for the partial digest problem. The algorithm is able to find all possible solutions for the input and works by traversing the solution tree with a breadth-first search in two stages and deleting all repeated subproblems. Two types of simulated data, random and Zhang, are used to measure the efficiency of the algorithm. We also apply the algorithm to real data for the Luciferase gene and the *E. coli* K12 genome.

**Conclusion:**

Our algorithm is a fast tool to find the exact solution for the partial digest problem. The percentage of improvement is more than 75% over the best known practical algorithm for the worst case. For large numbers of inputs, our algorithm is able to solve the problem in a suitable time, while the best known practical algorithm is unable.

**Electronic supplementary material:**

The online version of this article (doi:10.1186/s12859-016-1365-2) contains supplementary material, which is available to authorized users.

## Background

In 1970, Hamilton Smith discovered that long DNA molecules could be digested into a set of restriction fragments by the restriction enzyme HindII based on the occurrence of restriction sites with the sequences GTGCAC or GTTAAC [1]. Since that time, restriction enzymes have played a crucial role in many biological experiments, including genome editing [2], gene cloning [3], protein expression [4, 5] and genome mapping [6–11]. Restriction enzymes are commonly used to physically map genomes. In physical mapping, the restriction enzymes are used to cut a DNA molecule at restriction sites with the goal of identifying the locations of the restriction sites after digestion. Their positions in the genome are determined by analyzing the lengths of the digested DNA. Based on the experimental assumptions of digestion, there are two main types of digestions, a partial digest [9] and a double digest [10]. Constructing an accurate physical map following a partial digestion is a fundamental problem in genome analysis. In this work, we consider the partial digestion.

In a partial digestion experiment, one restriction enzyme is used to cut one or more target DNA molecules at several specific restriction site. The digestion results in a collection of short DNA fragments, and the lengths of these fragments are recorded in multiset *A*. Attempting to reconstruct the locations of the restriction sites in the target DNA molecules using multiset *A* is known as the Partial Digest Problem, PDP. Some modifications have been introduced into the partial digestion process to produce simplified variants of PDP. These variants include: the simplified partial digest problem, SPDP [12], the labeled partial digest problem, LPDP [13] and the probed partial digest problem, PPDP [14]. In this work, we consider the PDP.

Several algorithms [15–20] have been developed to solve the PDP. Some of these algorithms have short running times, but the solutions are not exact. These algorithms [15–17] are based on heuristic and approximation strategies. Other algorithms require a long running time in the worst case, but the solution is exact for any instance. These algorithms [18–20] are based on brute force or branch and bound strategies.

In this research paper, we describe an algorithm with a suitable run time that generates an exact solution for the PDP. The previous algorithms that yield an exact PDP solution can be divided into impractical and practical types. The impractical solutions are based on the brute force strategy and polynomial factorization [19]. The best known practical algorithm for PDP is the algorithm proposed by Skiena et al. [20], which is based on the branch and bound strategy. The algorithm is practical because the running time of the algorithm is *O* (*n* ^2^ *log n*) for an average case, while exponential amounts of time were required for the worst case.

### Problem formulation and related definitions

Before we give the formal definition of the PDP, we need the following related definitions.

**Definition 1** [21]**:** The **difference** of two multisets *D* and *L* denoted by *D*\*L* such that *D*\*L* = {*x*|*x* ∈ *D* and *C*(*D*\*L*, *x*) = *C*(*D*, *x*) − *C*(*L*, *x*) > 0}, where *C* (*D*, *x*) denotes the number of occurrences of element *x* ∈ *D* in *D*.

**Definition 2** [21]**:** The **sum** or (**disjoint union**) of two multisets *D* and *L* denoted by *D* ∪_+_ *L* such that *D* ∪_+_ *L* = {*x*|*x* ∈ *D* or *x* ∈ *L* and *C* (*D* ∪_+_ *L*, *x*) = *C* (*D*, *x*) + *C* (*L*, *x*)}.

**Definition 3** [22]**:** The differences of element *y* and a set *X* denoted by *Δ* (*y*, *X*) such that *Δ*(*y*, *X*) = {|*y* − *x*_*i*_| : *x*_*i*_ ∈ *X*}.

**Definition 4** [22]**:** The differences for a set *X*, denoted by *ΔX*, is a multiset such that: *ΔX* = {|*x*_*j*_ − *x*_*i*_|, 0 ≤ *i* < *j* ≤ *n* − 1}.

**Remark 1:** We can write *ΔX* in another form *ΔX* = _*i* = 0_^*n* − 1^ ∪_+_ *Δ* (*x*_*i*_, (*X*\*x*_*i*_)), where *X* = {*x*_0_, *x*_1_, …, *x*_*n* − 1_}.

**The Partial Digest Problem, PDP** [11]**:** Given a multiset of $$ N=\left(\begin{array}{c}\hfill n\hfill \\ {}\hfill 2\hfill \end{array}\right) $$ positive integers *D* = {*d*_0_, *d*_1_, *d*_2_, …, *d*_*N* − 1_}. Is there a set of *n* integers X = {*x*_0_, *x*_1_, *x*_2_, …, *x*_*n* − 1_} such that *ΔX* = *D* ?.

We also need two propositions. The first proposition is used to give another formula for the difference between three multiple sets. The formula will be used to prove the correctness of our proposed method. The second proposition is used to illustrate how to construct an example for the worst case, which leads to an exponential time for Skiena’s algorithm [23].

**Proposition 1:** Let *D*, *L* and *Z* be three multisets, then (*D*\*L*)\*Z* = *D*\(*L* ∪_+_ Z).

**Proposition 2** [23]: Let $0<\varepsilon <\frac{1}{12}n$, *A*_1_ = {1 − *nε*, …, 1 − 2*ε*, 1 − *ε*}, *A*_2_ = {*ε*, 2*ε*, …, *nε*}, *A*_3_ = {(*n* + 1)*ε*, (*n* + 2)*ε*, …, 2*nε*}, *A*_4_ = {(2*n* + 1)*ε*, (2*n* + 2)*ε*, …, 3*nε*}, *A*_5_ = {1 − 3*nε*, …, 1 − (2*n* + 2)*ε*, 1 − (2*n* + 1)*ε*} and *D* = *F* ∪ *G* where *F* and *G* are disjoint sets satisfying *F* ∪ G* = *A*_3_ and G* = {1 − *g*| ∀ *g* ∈ *G*}. Let *A* = *A*_1_ ∪ *A*_2_ ∪ *A*_4_ ∪ *A*_5_ ∪ *D* ∪ {0, 1}, we can choose *D* such that giving *ΔA* to Skiena’s algorithm will take it at least *Ω*(2^*n* − 1^) time to find *A*.

## Methods

In this section, we present three algorithms. The first one is the best previous practical algorithm, while the other two algorithms are the proposed algorithms.

### Best previous practical algorithm

The main goal of PDP is to reconstruct the elements, *x*_*i*_, from a multiset, *D*, of *N* = *n* (*n* − 1)/2 integers by finding a set *X* such that *ΔX* = *D*. The best known algorithm for solving the PDP is based on the branch and bound strategy. The main idea of this algorithm is to construct the set *X* incrementally. The algorithm is based on the depth-first search algorithm with two bounding conditions. We refer to this method as Algorithm BBd (*b*ranch and *b*ound based on *d*epth).

### Observation

**Fig. 1:**
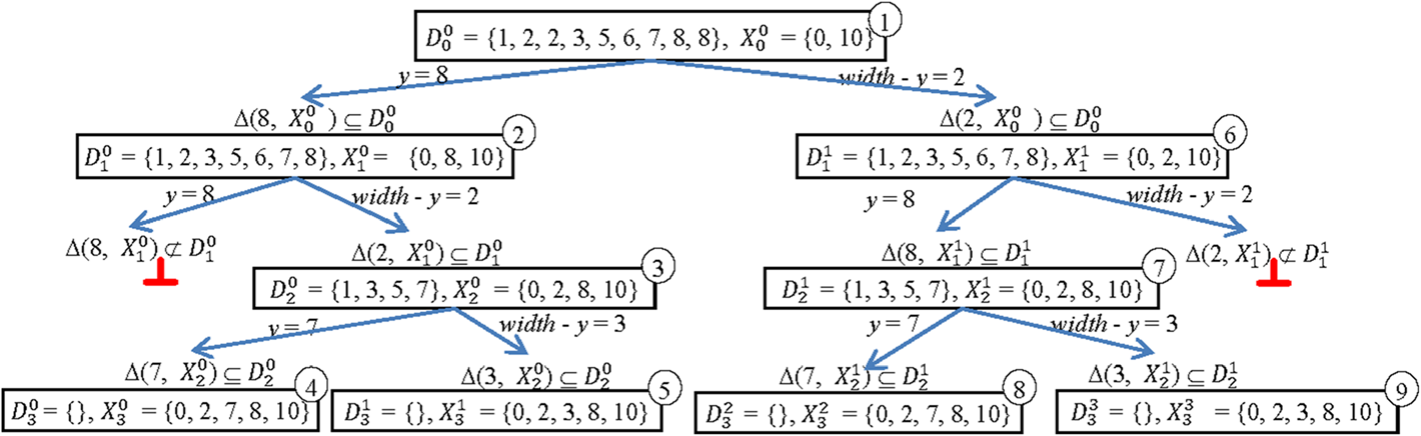
Tracing the BBd algorithm on *D* = {1, 2, 2, 3, 5, 6, 7, 8, 8, 10}

Figure 1 represents the execution of the algorithm BBd on *D* = {1, 2, 2, 3, 5, 6, 7, 8, 8, 10}. In the figure, we use the following notations.

i. Each node in the solution tree for the PDP represents a pair of sets (*D*_*k*_^*i*^, *X*_*k*_^*i*^) where the index *k* represents the level number, and the index *i* represents the node number in the level *k*.
ii. The number *t* inside the circle at the top right of each node represents the *t*-th calling for the procedure Place.
iii. The symbol “⊥” is used when the current node does not generate any new elements.

It is clear that at the level 0, the root contains the multiset *D*_*0*_^*0*^ = {1, 2, 2, 3, 5, 6, 7, 8, 8} and the set *X*_*0*_^*0*^ = {0, 10}. In general, *X*_*0*_^*0*^ = {0, *width*} and *D*_*0*_^*0*^ = *D* \ {*width*}, where *D* is the input of the PDP**.** Additionally, each node (*D*_*k*_^*j*^, *X*_*k*_^*j*^) at the level *k, k* > 0, of the solution tree generates at most two nodes at the level *k +* 1 as follows:

1. Add an element *y* to *X*_*k*_^*j*^ to generate *X*_*k* + 1_^*i*^ = *X*_*k*_^*j*^ ∪ {*y*}.
2. Remove the elements of *Δ* (*y*, *X*_*k*_^*j*^) from *D*_*k*_^*j*^ to generate *D*_*k* + 1_^*i*^ = *D*_*k*_^*j*^ \ Δ(*y*, *X*_*k*_^*j*^).

where *y* = Max (*D*_*k*_^*j*^) or *y* = *width* ‐ Max(*D*_*k*_^*j*^). We also observe from Fig. 1 the following:

1. There are two identical subproblems (*D*_*2*_^*0*^, *X*_*2*_^*0*^) and (*D*_2_^1^, *X*_2_^1^), such that *D*_2_^0^ = *D*_2_^1^ = {1, 3, 5, 7} and *X*_2_^0^ = *X*_2_^1^ = {0, 2, 8, 10}.
2. The two identical subproblems lead to two identical solutions, {0, 2, 7, 8, 10} and {0, 2, 3, 8, 10}.

### First proposed method

In this subsection, we propose an efficient method that reduces the running time of the PDP. The method is based on traversing the solution tree for the PDP using the breadth-first strategy instead of the depth-first strategy. We also consider the two bounding conditions that are used in algorithm BBd. Moreover, we remove all identical subproblems at the same level. The main steps of our method are as follows:

1. Build the solution tree for the PDP using the breadth-first strategy, level by level.
2. Before creating the nodes of the new level, we remove all repeated nodes existing in the current level such that any node appears only one time in the current level.

Algorithm BBb (*b*ranch and *b*ound based on *b*readth) shows the steps of our proposed method to solve the PDP. The input of the algorithm is the multiset *D* that consists of *n* (*n* − 1)/2 elements. Initially the algorithm starts with two sets and two lists. The two sets are *X*_0_^0^ = {0, *width*} and *D*_0_^0^ = *D* \ {*width*}. The two lists are *L*_*X*_ = {*X*_0_^0^} and *L*_*D*_ = {*D*_0_^0^}. In general, *L*_*D*_ and *L*_*X*_ represent the lists of sets, *D*_*k*_^*j*^ and *X*_*k*_^*j*^, respectively, at the current level, *k*, of the solution tree. The main step of the proposed algorithm is a while loop that represents the number of levels in the solution tree for the PDP. In each iteration *k* of the while loop, we will generate the elements of the next level, *k* + 1, by calling the procedure GenerateNextLevel (*L*_*D*_, *L*_*X*_, *S*), *k* ≥ 0. The inputs of the procedure are three lists of sets, *L*_*D*_, *L*_*X*_ and *S*, for the current level *k*. The outputs of the procedure are three lists of sets, *L*_*D*_, *L*_*X*_ and *S*, for the level *k* + 1.

The body of the procedure GenerateNextLevel consists of an initialization and a loop. In the initialization, we will use two auxiliary lists, *AL*_*D*_ and *AL*_*X*_, which contain the sets *D*_*k* + 1_^*j*^ and *X*_*k* + 1_^*j*^, respectively, for the next level in the solution tree. The initial value of the two lists is empty. The main loop in the GenerateNextLevel procedure represents the process of generating the elements of the next level and storing it in the two auxiliary lists *AL*_*D*_ and *AL*_*X*_. Each pair of sets, *D*_*k*_^*i*^ ∈ *L*_*D*_ and *X*_*k*_^*i*^ ∈ *L*_*X*_, will generate at most two pairs of sets, as follows:

(i) *D*_*k* + 1_^*j*^ = *D*_*k*_^*i*^\Δ (*y*, *X*_*k*_^*i*^) and *X*_*k* + 1_^*j*^ = *X*_*k*_^*i*^ ∪ {*y* } if the condition Δ (*y* , *X*_*k*_^*i*^) ⊆ *D*_*k*_^*i*^ is true.
(ii) *D*_*k* + 1_^*l*^ = *D*_*k*_^*i*^ \ Δ (*width* ‐ *y*, *X*_*k*_^*i*^) and *X*_*k* + 1_^*l*^ = *X*_*k*_^*i*^ ∪ {*width* ‐ *y* } if the condition Δ (*width* ‐ *y* , *X*_*k*_^*i*^) ⊆ *D*_*k*_^*i*^ is true.

The two sets, *X*_*k* + 1_^*j*^ and *D*_*k* + 1_^*j*^ and in a similar way, *X*_*k* + 1_^*l*^ and *D*_*k* + 1_^*l*^, will be added to the auxiliary lists *AL*_*X*_ and *AL*_*D*_, respectively if set *X*_*k* + 1_^*j*^ does not exist in list *AL*_*X*_.

**Fig. 2:**
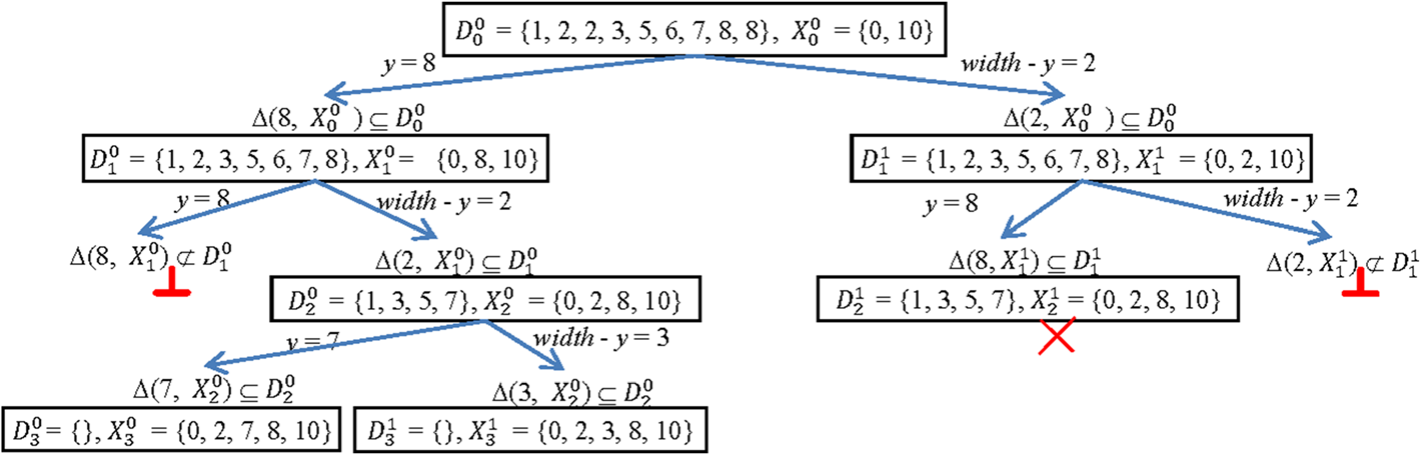
Tracing the BBb algorithm on *D* = {1, 2, 2, 3, 5, 6, 7, 8, 8, 10}

The main loop of the GenerateNextLevel procedure will terminate when list *L*_*D*_ is empty. This means that all of the elements of the current level *k* are replaced by new elements in the next level *k* + 1. In this case, we assign the lists *AL*_*D*_ and *AL*_*X*_ to *L*_*D*_ and *L*_*X*_, respectively. Figure 2 illustrates how this idea works.

Now, we investigate how to test the equality between two nodes (*D*_*k*_^*i*^, *X*_*k*_^*i*^) and (*D*_*k*_^*j*^, *X*_*k*_^*j*^) in the solution tree for the PDP. The test can be performed by comparing the equality between *D*_*k*_^*i*^ and *D*_*k*_^*j*^ and the equality between *X*_*k*_^*i*^ and *X*_*k*_^*j*^. The following theorems and corollary prove that the two nodes (*D*_*k*_^*i*^, *X*_*k*_^*i*^) and (*D*_*k*_^*j*^, *X*_*k*_^*j*^) are equal if the set *X*_*k*_^*i*^ is equal to the set *X*_*k*_^*j*^.

**Theorem 1:** Given a subproblem (*D*_*k*_^*i*^, *X*_*k*_^*i*^) in the solution tree for the PDP, the relation *D*_*k*_^*i*^ = *D* \ Δ*X*_*k*_^*i*^ is valid, where *k* ≥ 0, *D* is the input of the PDP, *D*_*k*_^*i*^ is a modified version of *D* produced by removing some of its elements and *X*_*k*_^*i*^ contains *k* + 2 elements of the candidate solution.

***Proof*** **:** We will prove the theorem using mathematical induction.

First, we prove that the relation is true at *k* = 0.

The algorithm starts by finding the maximum elements of the set *D*, *width*, and updates the value of the sets *D* and *X*. So, *X*_0_^0^ = {0, *width*} and *D*_0_^0^ = *D*\{*width*}. From the definition of *Δ*, we have, *ΔX*_0_^0^ = {*width*}. Therefore, *D*_0_^0^ = *D*\*ΔX*_0_^0^.

Second, we assume that the relation is true at *k* (i.e., *D*_*k*_^*i*^ = *D*\*ΔX*_*k*_^*i*^).

Third, we prove that the relation is true at *k* + 1.

From the algorithm, the set *D*_*k* + 1_^*j*^ can be constructed from a node at level *k*, say (*D*_*k*_^*i*^, *X*_*k*_^*i*^). Therefore, *D*_*k* + 1_^*j*^ = *D*_*k*_^*i*^ \ *Δ* (*y*, *X*_*k*_^*i*^) and *X*_*k* + 1_^*j*^ = *X*_*k*_^*i*^ ∪ {*y*}. This implies that *D*_*k* + 1_^*j*^ = (*D* \ *ΔX*_*k*_^*i*^)\*Δ*(*y*, *X*_*k*_^*i*^), because *D*_*k*_^*i*^ = *D* \ *ΔX*_*k*_^*i*^. Therefore, *D*_*k* + 1_^*j*^ = *D* \ (*ΔX*_*k*_^*i*^ ∪_+_ *Δ*(*y*, *X*_*k*_^*i*^)) (from Proposition 1). From Definition 4 and because *X*_*k* + 1_^*j*^ = {*y*} ∪ *X*_*k*_^*i*^, then *D*_*k* + 1_^*j*^ = *D* \ *ΔX*_*k* + 1_^*j*^.

**Theorem 2:** If there are two subproblems (*D*_*k*_^*i*^, *X*_*k*_^*i*^) and (*D*_*k*_^*j*^, *X*_*k*_^*j*^) such that *X*_*k*_^*i*^ = *X*_*k*_^*j*^, then *D*_*k*_^*i*^ = *D*_*k*_^*j*^.

***Proof*** **:** From Theorem 1, the following equations are valid *D*_*k*_^*i*^ = *D*\*ΔX*_*k*_^*i*^ and *D*_*k*_^*j*^ = *D*\*ΔX*_*k*_^*j*^, for the subproblems (*D*_*k*_^*i*^, *X*_*k*_^*i*^) and (*D*_*k*_^*j*^, *X*_*k*_^*j*^), respectively. Because *X*_*k*_^*i*^ = *X*_*k*_^*j*^, then *D*_*k*_^*i*^ = *D*\*ΔX*_*k*_^*i*^ = *D*\*ΔX*_*k*_^*j*^ = *D*_*k*_^*j*^.

**Corollary 1:** If there are two subproblems (*D*_*k*_^*i*^, *X*_*k*_^*i*^) and (*D*_*k*_^*j*^, *X*_*k*_^*j*^) such that *X*_*k*_^*i*^ = *X*_*k*_^*j*^, then (*D*_*k*_^*i*^, *X*_*k*_^*i*^) = (*D*_*k*_^*j*^, *X*_*k*_^*j*^).

### Final proposed algorithm

We proposed an enhanced version of algorithm BBb. The proposed algorithm improves the running time and storage of the BBb algorithm especially for the worst case. In the proposed algorithm, we try to reduce the memory consumption of the BBb algorithm without increasing its running time. The improved algorithm depends on the following two steps:

1. Building the solution tree for the PDP until a specific level α is reached by using the BBb algorithm.
2. For each node in the level α, building the remainder subtrees individually with the BBb algorithm.

**Fig. 3:**
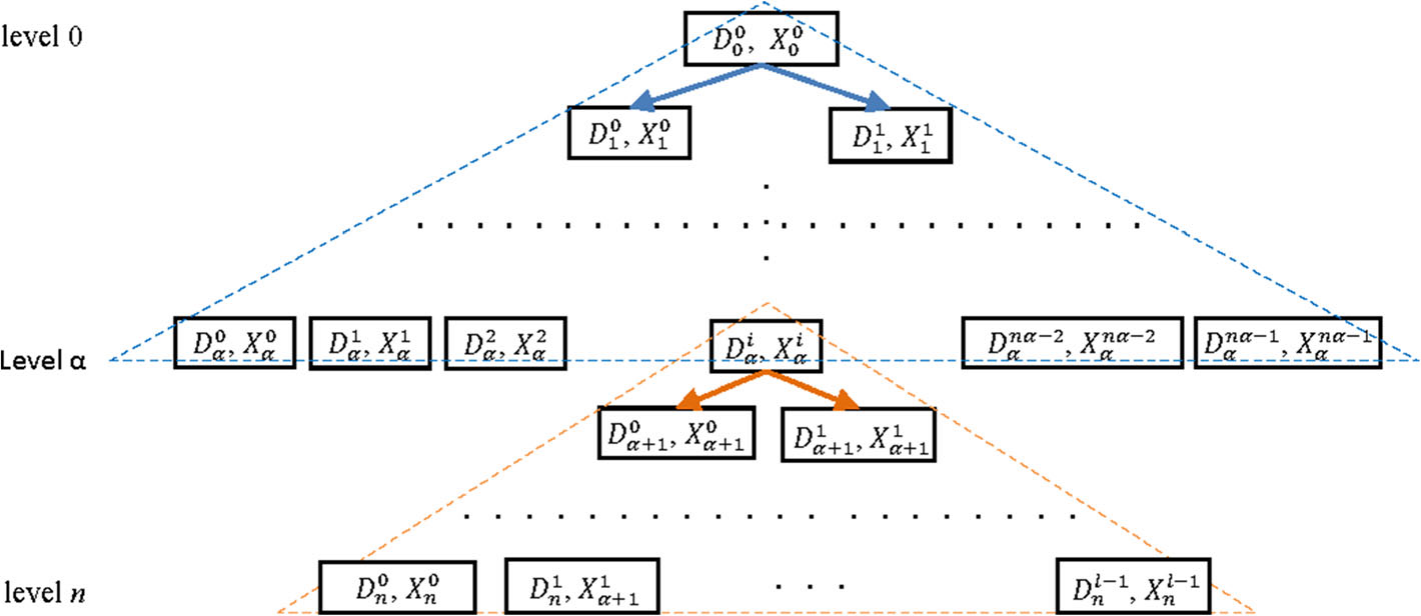
Strategy of the BBb2 algorithm

Figure 3 represents the idea described above for the proposed algorithm. We called the algorithm BBb2, *b*ranch and *b*ound based on *b*readth two times.

To determine the best value of α, we need to compute the memory complexity of the proposed algorithm for the worst case. Each node in level *k* will be replaced by at most two nodes in the level *k* + 1, 0 ≤ *k* < *α*. Therefore, the total number of nodes at level *α* is 2^*α*^. In each node, we store two sets, *D*_α_^*i*^ and *X*_α_^*i*^, of total size *O* (*n* ^2^). Hence, the total amount of storage necessary to reach the level *α* is *O* (*n* ^2^ 2^*α*^) memory for BBb2. In the second step of the BBb2 algorithm, we apply BBb on each node individually. The maximum number of remaining levels is *n* − *α* and the total amount of memory required for the second step for the worst case is *O* (*n* ^2^ 2^*n* − *α*^). Hence, the memory complexity of the BBb2 algorithm for the worst case is given by:

$$ M\left(\alpha \right)=O\;\left({n}^2\ {2}^{\alpha}\right)+O\;\left({n}^2\ {2}^{n-\alpha}\right) $$

Let *α*_*M*_ be the number of levels that lead to the minimum memory required by BBb2. The value of *α*_*M*_ can be computed by determining the number of levels, α, that minimizes the memory consumption *M*(*α*), for 0 ≤ *α* ≤ *n* − 1.The Find_α_*M*_ procedure computes the value of α_*M*_ for the instance *n* in *O*(*n*) time. The input of the procedure is the size of the multiset *D*, *N*, and the output of the procedure is α_*M*_. From the size of the multiset *D*, we can compute the value of *n* by solving the quadratic equation $\frac{n\;\left(n-1\right)}{2}=N$. The pseudocode of the Find_α_*M*_ procedure is as follows:

Procedure Find_α_*M*_ (*N*, α_*M*_)

Begin

1. $$ n=\frac{1+\sqrt{1+8N}}{2} $$
2. $$ \alpha = {\alpha}_M = 1 $$
3. $$ {M}_{min}=\kern0.37em {n}^2\left({2}^{\alpha }+{2}^{n-\alpha}\right) $$
4. for α = 2 to *n* do
5. $$ M={n}^2\left({2}^{\alpha }+{2}^{n-\alpha}\right) $$
6. if *M* < *M*_*min*_ then
7. $$ {M}_{min}=M $$
8. $$ {\alpha}_M = \alpha $$
9. end if
10. end for

End

In Algorithm BBb2, we start by finding the value of *α*_*M*_ and then we build the solution tree until the level *α*_*M*_ is reached by using the breadth-first strategy and deleting all similar elements. At the level *α*_*M*_, we have at most ${2}^{\alpha_M}$ elements. After obtaining these elements, we consider each node as a root and then expand this node using the BBb algorithm. For the worst case, the number of handled elements at levels α_*M*_ + 1, α_*M*_ +2, …,*n* are 2, 2^2^, …, ${2}^{n-{\alpha}_M}$ respectively, while the number of handled elements during execution of the BBb algorithm at levels α_*M*_ + 1, α_*M*_ + 2,…,*n* are ${2}^{\alpha_M+1}$, ${2}^{\alpha_M+2}$, …,2^*n*^ respectively. Therefore, BBb2 reduces the memory required by the BBb algorithm.

## Test methodology

In this section, we present the methodology that is used to evaluate the performance of the algorithms, BBd, BBb and BBb2, according to their running times and memory consumptions.

### Platform Specification

We implemented the algorithms on a Dual Octa-core processor machine (Intel Xeon E5-2690) with 128 GB RAM. Each processor has a 2.9 GHz speed with 20 MB of cache. The algorithms were implemented in C++ programs. The programs were compiled using g++ with the -O3 flag under 64-bit Red Hat Enterprise Linux 4.4. In the experiments, we used a single core only.

### Simulated data

We studied the performance of the three algorithms, BBd, BBb and BBb2, on different types of data and different sized data sets. We used two types of data described in previous studies. The first data set was a random data set (RD) [16], while the second data set is the Zhang data (ZD) [16, 17, 23]. The RD was used to measure the performance of the algorithm for an average case; while the ZD was used to measure the performance of the algorithm for the worst case. In terms of data set size, there are two factors that affect the performance of the algorithms for each data set. The first factor is the number of elements in the set *X*, *n*, while the second factor is the values of the *n* elements, *M* (maximum elements of *X*).

In the case of the RD, we assumed that there were *n* restriction sites in the DNA segments distributed randomly. Each input instance of the simulated data is a multiset *D* = Δ*X* such that the set *X* contains *n* positive numbers randomly selected and each number is less than or equal to *M.* In the ZD, the locations of the restriction sites were selected randomly according to Proposition 2 [23].

The values of *n* in the case of the RD are 100, 200, 300,… and 1000, while the values of *n* in the case of ZD are 15, 20, 25, 30,… and 90. The range of *n* for the ZD is small because the running time for the algorithm was greater than 1 day when *n* > 90. We also used different values of *M* as follows: *M* = n*q, where *q* = 10, 100, 1000 and 10,000.

### Running time and memory

For each value of *n*, we ran each algorithm 50 times with different inputs for the RD. For the ZD, we reduced this number to 20 due to the increased running time of the algorithms, especially BBd. The running time for each algorithm for a fixed *n* represents the average time for all instances studied. If the running time of an algorithm was greater than 24 h for an input instance then the algorithm was terminated. Therefore, the value of the algorithm for this input instance was omitted from the figures and the results.

We also measured the standard error of the mean (SEM) and coefficient of variation (CV) for the three algorithms for RD and ZD. Finally, we used a non-parametric statistical test which is Wilcoxon-signed-rank test [24] to determine if there are a significant differences between the three algorithms in running time or memory consumption on RD and ZD. We applied the Wilcoxon-signed-rank test, at a significance level of 0.05, to the following pairs: BBd & BBb, BBd & BBb2 and BBb & BBb2 algorithms with respect to running time and memory consumption for RD and ZD.

We measured the running time in seconds using a C++ function. We also measured the memory consumed by the algorithm in megabytes using the Linux command *top*.

## Results and discussion

**Fig. 4:**
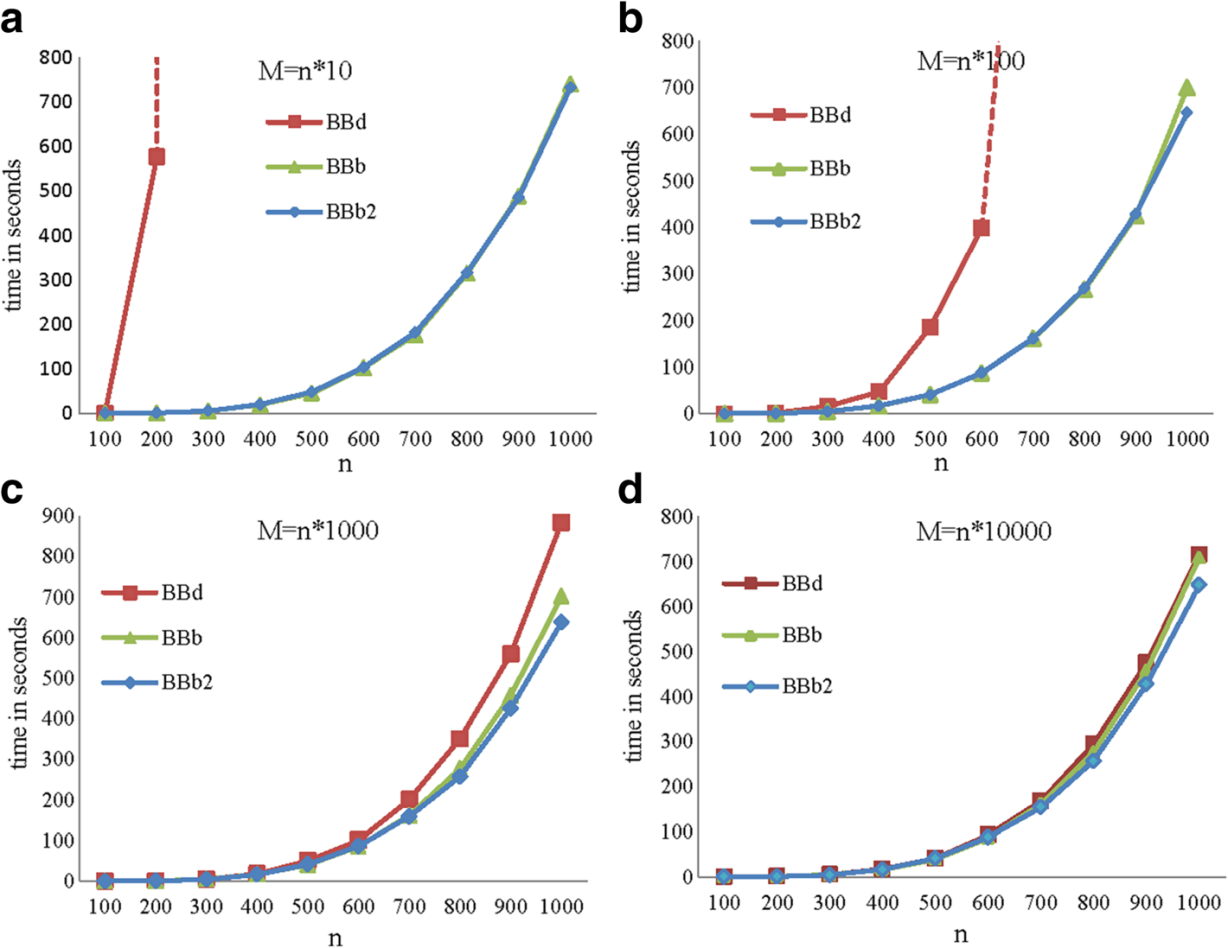
Running times of the BBd, BBb and BBb2 algorithms for the RD. The line for BBd algorithm is not complete because the running time is greater than the maximum value of y-axis or 24 h

**Fig. 5:**
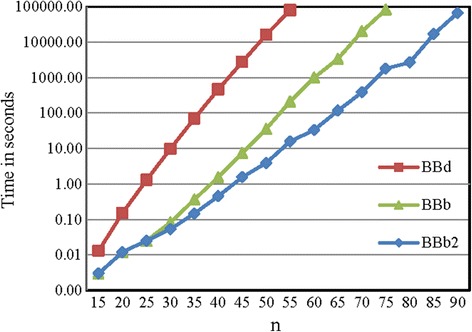
Running times of the BBd, BBb and BBb2 algorithms for the ZD. The values on the y-axis are in log-scale

The results from measuring the running times of the three algorithms on the simulated data are shown in Fig. 4 and (Additional file 1: Figure F1) for the RD and Fig. 5 for the ZD.

In the case of the RD, the results showed that the running times of the proposed algorithms, BBb and BBb2, were less than the running time of BBd for all values of *n* and *M* as shown in Fig. 4. For large values of *n* and small values of *q*, the BBd algorithm required a large amount of time to find a solution, while the proposed algorithms, BBb and BBb2, found the solution in a suitable time. For example, in the case of *n* ≥ 300 and *M* = *n* * 10, the running time for the BBd algorithm was greater than 24 h, while both proposed algorithms found the solution in time less than 13 min, as shown in Fig. 4a and b. However, the difference in running times between the BBd algorithm and the proposed algorithms, BBb and BBb2, decreased with increasing values of *M*. This behavior is attributed to the probability of repeated subproblems decreasing with increasing values of *M*. Therefore, both algorithms, BBb and BBb2, spend a large amount of time checking for the repetition of elements with a low probability of repetitions. Additionally, for small *M* values the probability of repeated subproblems is high, thereby increasing the running time of the BBd algorithm. If we fixed the value of *n*, the running time for all algorithms decreased with increasing *M* values as shown in (Additional file 1: Figures F1 a-c). Both proposed algorithms behaved similarly with increasing *M* values. We also observed that the difference in the running times for two successive values of *M* is relatively small for both proposed algorithms (especially when *M* is large) as shown in (Additional file 1: Figures F1 b and c). On the other hand, the difference in the running times for the BBd algorithm for two successive values of *M* is large when *M* = *n* * 100 and *M* = *n* * 1000 as shown in (Additional file 1: Figure F1a). Finally, we found that there was little difference, increasing or decreasing, between the running times of BBb and BBb2 for all values of *n* and *M*. In general, with large values of *n* and *M*, the BBb2 algorithm was faster than the BBb algorithm. For example, in the case of *n* ≥700 and *M* = *n* * *q* and *q* = 1000 and 10,000, BBb2 was slightly better than BBb.

For the ZD, there was no change in the running time when we used different values of *M* for the three algorithms. Therefore, we used the ZD with the *M* value fixed at *M* = n*1000. The consistent performance of the algorithms using the ZD with different values of *M* was due to the systematic selection of the elements of *A* according to Proposition 2.

The performance of the three algorithms for the Zhang data set instances is given in Fig. 5. We observed that the running time of BBb2 was less than BBb and BBb was less than BBd for all instances. The percentage of running time improvement for BBb and BBb2 with respect to BBd increased with increasing *n*. In our studied cases, the running time was improved by at least 75%. Moreover, the BBd and BBb algorithms cannot solve any instances with *n* ≥ 60 and *n* ≥ 80 in time less than 24 h respectively. In the other side, the BBb2 algorithm solves any instance with $n\le 90$ in time less than 24 h.

The improved running time of BBb algorithm can be attributed to its reduction of the number of subproblems handled at the levels *α*_*M*_ + 1, *α*_*M*_ + 2,…,*n* in the solution tree for the PDP. Therefore, the time spent checking subproblem repetition is reduced.

The results of measuring SEM and CV for the running time of the three algorithms are shown in (Additional file 2: Tables S1–S5) for RD and ZD. The results show that the values of SEM and CV for BBd algorithm were very large compared to the values of SEM and CV for BBb and BBb2 algorithms in case of RD. For the ZD, the values of SEM and CV of BBb and BB2 algorithms were less than the values of SEM and CV of BBd algorithm in the most instances. Moreover, the application of Wilcoxon-signed-rank test shows that there was a significant difference between the following pairs of the algorithms as shown in (Additional file 2: Tables S6–S14):

i. BBd and BBb in case of RD and ZD.
ii. BBd and BBb2 in case of RD and ZD.
iii. BBb and BBb2 in case of ZD.

**Fig. 6:**
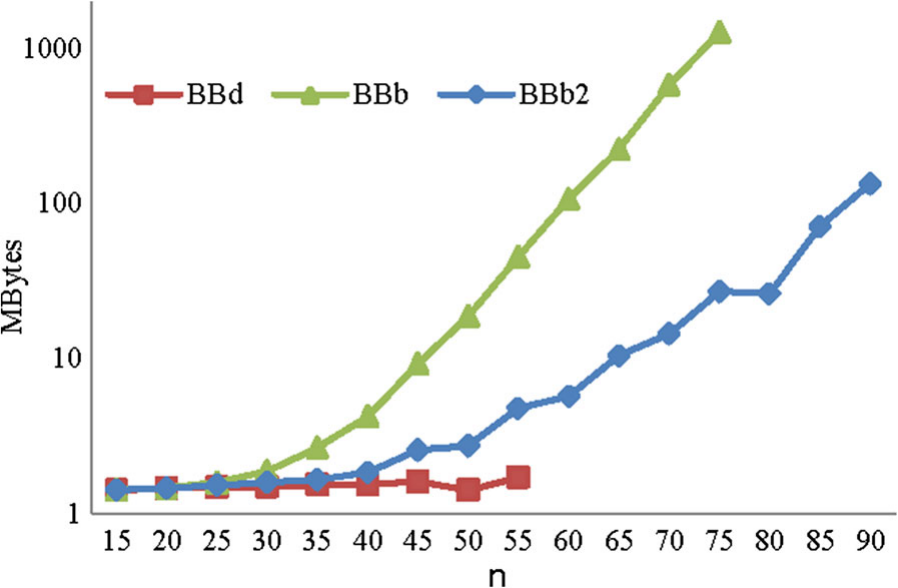
Memory consumed of BBd, BBb, and BBb2 algorithms on ZD. The values on the y-axis are in log-scale

We also evaluated the algorithms in terms of their memory consumptions. The results of measuring the memory consumed by the three algorithms on the simulated data are shown in (Additional file 3: Figures F2 a–d) for the RD and Fig. 6 for the ZD. For the RD, the results show that for small values of *M* such as *M* = *n* * 10 and *n* * 100, the BBb and BBb2 algorithms consumed less memory than the BBd algorithm. In the case of large *M* values, the BBd algorithm consumed less memory than the BBb and BBb2 algorithms, but small *M* values increased the number of repeated subproblems. Thus, the number of elements in each level is large for the BBd algorithm. Therefore, the BBd algorithm consumed more memory than the two proposed algorithms. In general, the memory consumption of the BBb algorithm is a little better than that of the BBb2 algorithm.

For the ZD, less memory was consumed by BBb2 than BBb for all instances. So, the BBb2 algorithm significantly reduced the memory required by the BBb algorithm. The percentage of improvement in memory consumption of BBb2 compared to BBb increases as *n* increases. In general, the memory required by the BBd algorithm is less than the BBb and BBb2 algorithms.

The results of measuring SEM and CV for the memory of the three algorithms are shown in (Additional file 4: Tables S15–S19) for the RD and the ZD. The results show that there were differences, decreasing and increasing, in the values of SEM and CV of the three algorithms for the RD. For the ZD, the values of SEM and CV of BBd algorithm were less than the proposed algorithms in the most instances. Moreover, the application of Wilcoxon-signed-rank test, see (Additional file 4: Tables S20-S28), shows that there was a significant difference between the following pairs of the algorithms in the following cases:

i. BBd and BBb on ZD.
ii. BBd and BBb2 on ZD.
iii. BBb and BBb2 on ZD.

## Real data

We tested the final proposed BBb2 algorithm on real digestion experiments and simulated digestion experiments.

### Luciferase gene

We extracted the data from a partial digestion of the luciferase gene [25] of length 2009. The partial digestion was performed with the enzyme *TaqI*, which cuts the gene at the *tcga* sequence motif. The output of the partial digestion process is the multiset $D$ consisting of the distances between the *tcga* locations on the luciferase gene, which is *D* = {9, 30, 100, 170, 293, 302, 393, 402, 462, 562, 632, 732, 855, 864, 945, 954, 975, 984, 1025, 1034, 1247, 1277, 1347, 1377, 1809, 1839, 1979, 2009}. Our proposed algorithms take the multiset *D* as input and output two solutions in the set *S* = {{0, 170, 632, 732, 1025, 1034, 1979, 2009}, {0, 30, 975, 984, 1277, 1377, 1839, 2009}}. The solution *X* = {0, 30, 975, 984, 1277, 1377, 1839, 2009}, represents the solution for the real data. This means that the map of *tcga* on luciferase gene at the locations 30, 975, 984, 1277, 1377, and 1839.

### *E. Coli* K12 genome digestion simulation

We also tested our proposed algorithms with a simulated partial digestion experiment using the *E. coli* K12 genome version 3 (NC_000913.3, downloaded 11 March 2016) and a set of restriction enzymes (https://www.en.wikipedia.org/wiki/Restriction_enzyme). The size of the genome is 4,641,652 bp. For each restriction enzyme, we simulated the partial digestion experiment by cutting the *E. coli* K12 genome at all occurrences of the corresponding restriction site. Then, we recorded the lengths of *N* cut fragments in *D*. The restriction enzymes, restriction sites, the number of cut fragments (*N*), *n*, times and memory consumptions are shown in Table 1. Finally, we applied the final proposed algorithm to the extracted sets and in all cases the outputs contained the correct set of restriction site positions. It is clear that from Table 1, the running time and memory consumed of the algorithm increases with increase the value of *n* and *N*.

**Table 1:** The Performance of the BBb2 algorithm for the restriction enzymes used in this study

Restriction enzyme	Restriction site	*N*	*n*	Time (Seconds)	Memory (MB)
NotI	GCGGCCGC	780	40	0.007	0.2
XbaI	TCTAGA	1891	62	0.017	3.6
SmaI	CCCGGG	103,285	455	56.2	8.9
BamHI	GGATCC	135,460	521	72	11.7
HindIII	AAGCTT	170,820	585	138.6	13.6
EcoRI	GAATTC	228,826	677	360	15.5
PvuII	CAGCTG	1,599,366	1789	24,012	98.8
EcoRV	GATATC	1,999,000	2000	42,840	979

*Abbreviations*: *N* is the number of cut fragments, *n* is the number of restriction sites

## Conclusions

In this paper, we addressed the PDP and proposed two algorithms, BBb and BBb2. In the BBb algorithm, we built the solution tree for the PDP in the breadth-first manner instead of the depth-first manner taking into consideration two conditions of pruning and deleting all repeated subproblems in the same level. The BBb solves many instances which are not solved by BBd in time less than 24 h. The main disadvantage of BBb is that the memory consumed grows exponentially. In BBb2, we reduced the memory required by BBb by solving the problem using two stages, with each stage working in the breadth-first manner. We also determined the number of the levels, which leads to reduced memory consumption without increasing the running time.

We measured the efficiency of the proposed algorithm compared to the best known practical algorithm on the basis of time and memory consumption. In the evaluation, we considered the following parameters: (1) types of data, RD and ZD; (2) value of *n*; and (3) value of *M*. In the case of running time, the BBb2 algorithm is faster than other algorithms. The efficiency increased when the ZD was used. In the case of memory, the BBd algorithm consumed less memory than other algorithms, but the running time was very slow especially for the ZD. Finally, we applied the BBb2 algorithm on Luciferase gene and the *E. coli* K12 genome.

## Additional files

Additional file 1: Supplementary figure. Figure F1 a–c represents the behavior of the running time for BBd, BBb, and BBb2 algorithms with different values of M and fixed value of n. The values on the y-axis are in log-scale. In Figure a, the x-axis does not include the value of M = n * 10, because the running time is greater than 24 h. (PDF 8 kb)
Additional file 2: Supplementary data. Calculation of the Standard Error of Mean (SEM), Coefficient of Variation (CV), and Wilcoxon Signed-Rank test for the running time. **Tables S1-S5**. in Sheet 1 and 2, represent the calculation of SEM and CV for the running time of BBd, BBb, and BBb2 algorithms in case of random data and Zhang data respectively. **Tables S6–S8**. in Sheet 3, represent the Wilcoxon Signed-Rank test between BBd and BBb algorithms for the running time of Random and Zhang data. **Tables S9–S11**. in Sheet 4, represent the Wilcoxon Signed-Rank test between BBb and BBb2 algorithms for the running time of Random and Zhang data. **Tables S12–S14**. in Sheet 5, represent the Wilcoxon Signed-Rank test between BBd and BBb2 algorithms for the running time of Random and Zhang data. (XLS 168 kb)
Additional file 3: Supplementary figure. Figure F2 a–d represents the memory consumed for BBd, BBb, and BBb2 algorithms on random data. (PDF 88 kb)
Additional file 4: Supplementary data. Calculation of the Standard Error of Mean (SEM), Coefficient of Variation (CV), and Wilcoxon Signed-Rank test for the memory. **Tables S15-S19**. in Sheet 1 and 2, represent the calculation of SEM and CV for the memory consumption of BBd, BBb, and BBb2 algorithms in case of random data and Zhang data respectively. **Tables S20–S22**. in Sheet 3, represents the Wilcoxon Signed-Rank test between BBd and BBb algorithms for the memory consumption of Random and Zhang data. **Tables S23–S25**. in Sheet 4, represent the Wilcoxon Signed-Rank test between BBb and BBb2 algorithms for the memory consumption of Random and Zhang data. **Tables S26-S28**. in Sheet 5, represent the Wilcoxon Signed-Rank test between BBd and BBb2 algorithms for the memory consumption of Random and Zhang data. (XLS 167 kb)

## References

[1] Pevzner P (1995). DNA physical mapping and alternating eulerian cycles in colored graphs. Algorithmica.

[2] Baker M (2012). Gene-editing nucleases. Nat methods.

[3] Sambrook J, Fritsch EF, Maniatis T (1989). Molecular cloning: a laboratory manual.

[4] Liu Z, Ping-Chang Y (2012). Construction of pET-32 α (+) vector for protein expression and purification. N am j med sci.

[5] He X, Hull V, Thomas JA, Fu X, Gidwani S, Gupta YK, Black LW, Xu SY (2015). Expression and purification of a single-chain type IV restriction enzyme Eco94GmrSD and determination of its substrate preference. Sci rep.

[6] Narayanan P. Bioinformatics: A primer. New Age International. 2005. ISBN 10: 8122416101, ISBN 13: 9788122416107.

[7] Kalavacharla V, Hossain K, Riera-Lizarazu O, Gu Y, Maan SS, Kianian SF (2009). Radiation hybrid mapping in crop plants. Adv agron.

[8] Dear PH. Genome mapping. eLS 2001. John Wiley & Sons. doi:10.1038/npg.els.0001467.

[9] Błażewicz J, Formanowicz P, Kasprzak M, Jaroszewski M, Markiewicz WT (2001). Construction of DNA restriction maps based on a simplified experiment. Bioinformatics.

[10] Paliswiat B, Pryputniewicz P (2004). On the complexity of the double digest problem. Control cybern.

[11] Cieliebak M, Eidenbenz S, Penna P (2003). Noisy data make the partial digest problem NP-hard. Lect notes comput cci.

[12] Blazewicz J, Burke EK, Kasprzak M, Kovalev A, Kovalyov MY (2007). Simplified partial digest problem: enumerative and dynamic programming algorithms. IEEE/ACM trans comput biol bioinform.

[13] Pandurangan G, Ramesh H (2002). The restriction mapping problem revisited. J comput syst sci.

[14] Karp RM, Newberg LA (1995). An algorithm for analysing probed partial digestion experiments. Comput appl biosci.

[15] Dakic T: On the turnpike problem. PhD thesis, Simon Fraser University 2000, ISBN:0-612-61635-5.

[16] Nadimi R, Fathabadi HS, Ganjtabesh M (2011). A fast algorithm for the partial digest problem. Jpn j ind appl math.

[17] Ahrabian H, Ganjtabesh M, Nowzari-Dalini A, Razaghi-Moghadam-Kashani Z (2013). Genetic algorithm solution for partial digest problem. Int j bioinform res appl.

[18] Rosenblatt J, Seymour PD (1982). The structure of homometric sets. SIAM j algebraic discrete meth.

[19] Lemke P, Werman M (1988). On the complexity of inverting the autocorrelation function of a finite integer sequence, and the problem of locating n points on a line, given the (nC2) unlabelled distances between them. Technical report # 453.

[20] Skiena SS, Smith WD, Lemke P. Reconstructing sets from interpoint distances. SCG '90 Proceedings of the sixth annual symposium on Computational geometry, 332–9.

[21] Syropoulos A. Mathematics of multisets. WMP '00 Proceedings of the Workshop on Multiset Processing: Multiset Processing, Mathematical, Computer Science, and Molecular Computing Points of View. 2000;347–58.

[22] Jones NC, Pevzner P. An introduction to bioinformatics algorithms. Chapter 4, 83-123, MIT press 2004.

[23] Zhang Z (1994). An exponential example for a partial digest mapping algorithm. J comput biol.

[24] Woolson RF. Wilcoxon Signed‐Rank Test. Wiley encyclopedia of clinical trials 2008. doi:10.1002/9780471462422.eoct979.

[25] Devine JH, Kutuzova GD, Green VA, Ugarova NN, Baldwin TO (1993). Luciferase from the east European firefly luciola mingrelica: cloning and nucleotide sequence of the cDNA, overexpression in Escherichia coli and purification of the enzyme. Biochim biophys acta.

