# Supplementary figures and images for: A fast exact sequential algorithm for the partial digest problem
Source: BMC Bioinformatics. 2016 Dec 22;17(Suppl 19):510. doi: 10.1186/s12859-016-1365-2 (PMC5259970; doi:10.1186/s12859-016-1365-2)

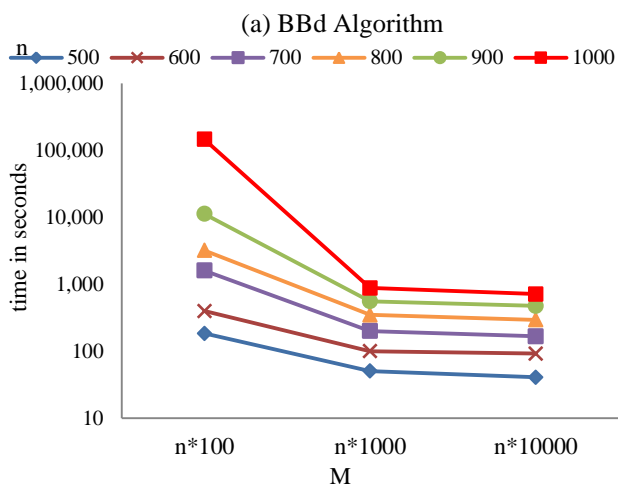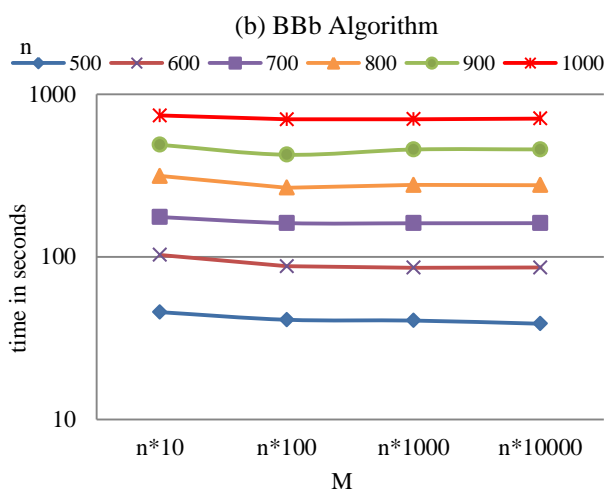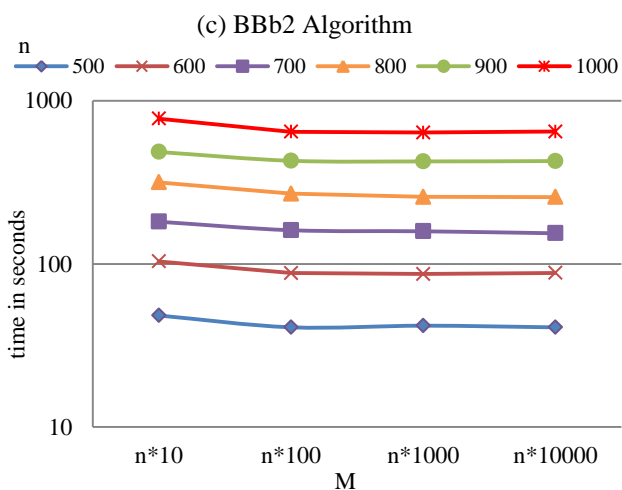

Figure F1: Running time of BBd, BBb, and BBb2 algorithms with different values of  $M$ .

Supplement: Additional file 1: — Supplementary figure. Figure F1 a–c represents the behavior of the running time for BBd, BBb, and BBb2 algorithms with different values of M and fixed value of n. The values on the y-axis are in log-scale. In Figure a, the x-axis does not include the value of M = n * 10, because the running time is greater than 24 h. (PDF 8 kb) [file 12859_2016_1365_MOESM1_ESM.pdf]

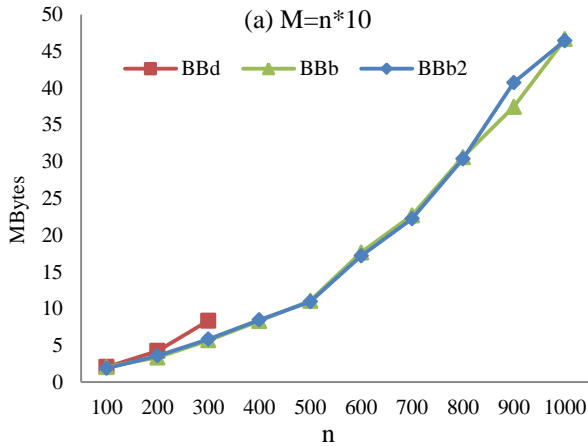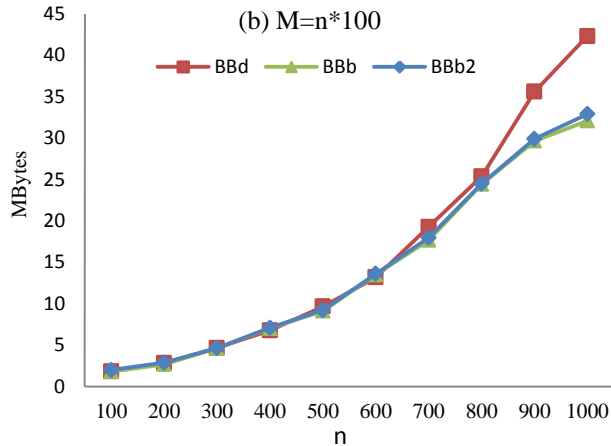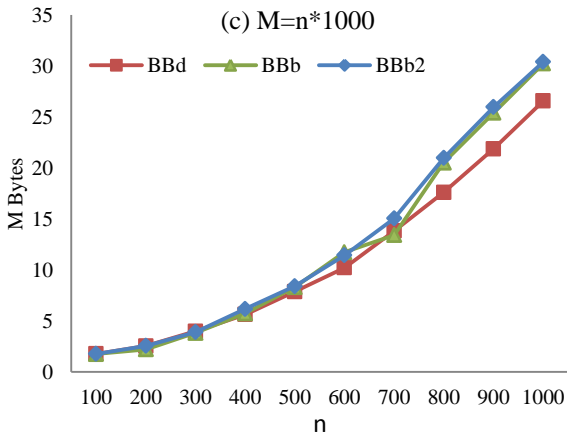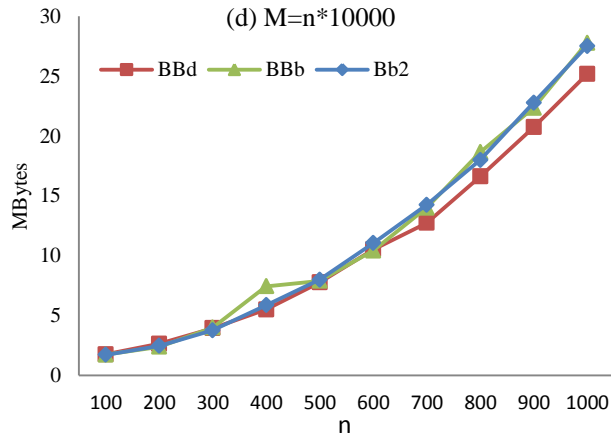

Figure F2: Memory consumed of BBd, BBb, and BBb2 algorithms on RD.

Supplement: Additional file 3: — Supplementary figure. Figure F2 a–d represents the memory consumed for BBd, BBb, and BBb2 algorithms on random data. (PDF 88 kb) [file 12859_2016_1365_MOESM3_ESM.pdf]
